# Glucocorticoid Therapy of Multiple Sclerosis Patients Induces Anti-inflammatory Polarization and Increased Chemotaxis of Monocytes

**DOI:** 10.3389/fimmu.2019.01200

**Published:** 2019-05-29

**Authors:** Henrike J. Fischer, Tobias L. K. Finck, Hannah L. Pellkofer, Holger M. Reichardt, Fred Lühder

**Affiliations:** ^1^Institute for Neuroimmunology and Multiple Sclerosis Research, University Medical Center Goettingen, Göttingen, Germany; ^2^Institute for Cellular and Molecular Immunology, University Medical Center Goettingen, Göttingen, Germany; ^3^Department of Neurology, University Medical Center Goettingen, Göttingen, Germany

**Keywords:** multiple sclerosis, methylprednisolone therapy, monocytes, M2 polarization, chemokines

## Abstract

Multiple Sclerosis (MS) is an autoimmune disease of the central nervous system (CNS), characterized by the infiltration of mononuclear cells into the CNS and a subsequent inflammation of the brain. Monocytes are implicated in disease pathogenesis not only in their function as potential antigen-presenting cells involved in the local reactivation of encephalitogenic T cells but also by independent effector functions contributing to structural damage and disease progression. However, monocytes also have beneficial effects as they can exert anti-inflammatory activity and promote tissue repair. Glucocorticoids (GCs) are widely used to treat acute relapses in MS patients. They act on a variety of cell types but their exact mechanisms of action including their modulation of monocyte function are not fully understood. Here we investigated effects of the therapeutically relevant GC methylprednisolone (MP) on monocytes from healthy individuals and MS patients *in vitro* and *in vivo*. The monocyte composition in the blood was different in MS patients compared to healthy individuals, but it was only marginally affected by MP treatment. In contrast, application of MP caused a marked shift toward an anti-inflammatory monocyte phenotype *in vitro* and *in vivo* as revealed by an altered gene expression profile. Chemotaxis of monocytes toward CCL2, CCL5, and CX3CL1 was increased in MS patients compared to healthy individuals and further enhanced by MP pulse therapy. Both of these migration-promoting effects were more pronounced in MS patients with an acute relapse than in those with a progressive disease. Interestingly, the pro-migratory GC effect was independent of chemokine receptor levels as exemplified by results obtained for CCR2. Collectively, our findings suggest that GCs polarize monocytes toward an anti-inflammatory phenotype and enhance their migration into the inflamed CNS, endowing them with the capacity to suppress the pathogenic immune response.

## Introduction

Multiple Sclerosis (MS) is an inflammatory autoimmune disease of the central nervous system (CNS) involving different types of immune cells including T cells, B cells, and monocytes. The most common disease course is characterized by acute relapses with complete or incomplete remission. This relapsing-remitting phenotype (RRMS) is observed in the majority of the MS patients, with young adults being most affected. RRMS can convert into a secondary-progressive form (SPMS) later in life, which is characterized by progressive worsening of the disease with or without additional relapses. The hallmark of the third form of MS, termed primary-progressive (PPMS), is a continuous worsening of the symptoms without intermittent improvements (1). Although numerous new drugs have been developed within the last decade, the most widely used treatment of acute relapses is still high-dose methylprednisolone (MP) pulse therapy to which most patients respond well, resulting in an amelioration of symptoms within a few days (2). Patients suffering from SPMS and PPMS are also treated with MP pulse therapy in the case that the disease is not stable. Mechanistically, various activities of glucocorticoids (GCs) affecting immune cells but also non-hematopoietic cell types are discussed (3, 4). Furthermore, the GC response was recently reported to be highly cell type-specific, both in magnitude and even direction of transcriptional regulation (5). In the context of MS therapy it is believed that patients profit most from direct or indirect dampening effects on T cells. It has been reported that GCs down-regulate expression levels of pro-inflammatory cytokines and adhesion molecules required to pass the blood-brain barrier (BBB). They also promote apoptosis induction in immune cells, inhibit T cell activation, and additionally exert inhibitory effects on inflammatory mediators such as nitric oxide (NO) (6). Our own preclinical studies using the animal model experimental autoimmune encephalomyelitis (EAE) further revealed that T cells are the major target cells of free administered GCs (7, 8). However, effects on myeloid cells were also shown to be crucial if GC were encapsulated in liposomes (9) or nanoparticles (10). In addition, we found that altered T cell migration along chemokine gradients was a mechanism accounting for the therapeutic activity of GCs in the treatment of neuroinflammation, whereas apoptosis induction in T cells unexpectedly turned out to be of minor importance (11).

T cells are the target of most current immunotherapies for MS patients, highlighting the importance of this cell population for the pathogenesis of MS. Nevertheless, myeloid cells including monocytes play important roles for innate immune responses and indirectly also influence adaptive immune responses by serving as antigen-presenting cells, and with both functions they also play a crucial role in MS and EAE (12). They are found in CNS lesions in EAE and MS and often outnumber infiltrating T cells. In animal models it has been shown that monocytic infiltration contributes to disease progression (13), and monocyte-derived macrophages are key players in the reactivation of infiltrating T cells (14). Consequently, elimination of macrophages (15–17) or selective depletion of CCR2^+^ Ly-6C^hi^ monocytes (18) reduced CNS inflammation. Alterations in the composition of monocyte subpopulations in the peripheral blood and cerebrospinal fluid (CSF) of MS patients have been reported as well, thus further highlighting their substantial role in human neuroinflammation (19).

In humans, monocytes are a heterogeneous cell population, constituting ~10% of total leukocytes in the blood. They have a short life span and evolve in three different subsets: the most prevalent being CD14^++^CD16^−^ classical (or inflammatory) monocytes, CD14^++^CD16^+^ intermediate state monocytes, and CD14^+^CD16^++^ non-classical monocytes (20). They can give rise to macrophages that encompass a dynamic spectrum of phenotypes with classical or M1 macrophages (producing IL-12, IL-1β, NO and reactive oxygen species, and acting in a pro-inflammatory fashion) and alternatively activated or M2 macrophages (expressing CD163, CD206, Arg1, and acting in an anti-inflammatory fashion) being the extreme ends of this spectrum (21). M2 myeloid cells were found to contribute to an improvement of autoimmune diseases such as MS and EAE (22–25). Similarly, skewed proportions of the different monocyte subsets have been reported for many human inflammatory and autoimmune diseases. For instance, in rheumatoid arthritis, systemic lupus erythematodes, sepsis, uveitis and sarcoidosis, intermediate-state monocytes were expanded (26–31). In contrast, data for MS patients concerning monocyte subsets are less consistent (19, 32, 33). Classical pro-inflammatory CD14^++^CD16^−^ monocytes are recruited to the CNS in response to CCL2. Non-classical CD14^+^CD16^++^ monocytes, however, are not necessarily beneficial in the context of MS. Namely, it has been shown that the latter cells can adhere to the endothelium and help T cells to extravasate at the site of inflammation and thereby contribute to MS pathology. Accordingly, they are found in active and demyelinating lesions and the CSF (33). Beyond their disease-promoting activity, however, monocytes are also able to dampen inflammation depending on their subtype and status.

The influence of different drugs used in the long-term treatment of MS such as glatiramer acetate (23), dimethyl fumarate (34), or fingolimod (35) on myeloid cell function has been intensively investigated. In contrast, effects of GCs on myeloid cells in the context of MS are less clear. Treatment of monocytes with GCs *in vitro* induces a stable anti-inflammatory gene expression profile (36). Consequently, such monocytes interfere with T-cell-mediated inflammation *in vivo*, where they were shown to directly suppress the secretion of IL-17 and IFNγ without inducing a direct Th2 shift. Additionally, treatment with GCs enables monocytes to induce regulatory T cells (Treg) at the site of inflammation (37, 38).

Here we investigate the influence of MP—it being the most widely applied GC in MS therapy—on human monocytes from healthy individuals and MS patients *in vitro* and *ex vivo*. We found evidence that monocyte polarization becomes skewed toward the M2 phenotype by MP treatment and that the migration of monocytes along chemokine gradients is increased without any significant changes in the level of their respective receptors. These findings suggest that GCs also exert their beneficial effects on MS bouts by tuning monocyte function and not necessarily solely by suppressing T cells.

## Materials and Methods

### Patients

Thirty patients with established diagnosis of MS according to the McDonald Criteria revised in 2017 were included in the current study (14 RRMS, 8 SPMS, 8 PPMS). All patients received high-dose MP (1,000 mg) intravenously on three consecutive days according to medical indication (due either to MS relapse or progressive worsening of neurologic symptoms in patients with progressive MS). Peripheral blood was drawn in Li-Heparin monovettes (Sarstedt, Nürnbrecht, Germany) before and 24 h after the first injection of MP. Due to the small volume of blood that could be obtained from each patient and due to the sometimes limited recovery of blood after MP therapy, not all types of analyses were performed for every patient. The number of patients included in each experiment is therefore indicated in the figure legends.

Information about MS patients included in this study (disease subtype, age, gender, severity of clinical symptoms as assessed by the Expanded Disability Score Scale (EDSS), acute relapse, disease duration, treatment) are summarized in Table 1. SPMS patients that were treated with MP due to an acute relapse (Table 1) were combined with the RRMS group and collectively referred to as “MS patients with acute relapse.” In contrast, SPMS patients without an acute relapse (Table 1) were combined with the PPMS group and referred to as “MS patients with progressive disease.” In addition, 24 healthy donors (age and gender summarized in Table 1) were included. The investigations were conducted according to the *Declaration of Helsinki* and national and international guidelines. The study was approved by the local ethics committee of the University Medical Center Göttingen. Informed written consent was obtained from each subject prior to the collection of blood.

**Table 1:** Summary of the characteristics of patients and healthy individuals included in the study.

	**Healthy individuals**	**RRMS**	**SPMS**	**PPMS**
Number	24	14	8	8
Age (years ± SD)	29.4 (8.8)	39.4 (8.9)	53.1 (7.5)	57.1 (10.2)
Females, number (%)	11 (45.8)	7 (50)	5 (62.5)	6 (75)
Mean EDSS score (±SD)	n.a.	2.54 (1.05)	6.12 (1.21)	5.5 (1.23)
Disease duration (Mean ± SD)	n.a.	4.79 (4.92)	22.63 (13.57)	12.5 (3.2)
Acute relapse, number (%)	n.a.	13 (92.8)	4 (50)	–
Disease modifying therapy, number (%)	n.a.			–
Fingolimod		4 (28.6)	–	–
IFNβ		4 (28.6)	–	–
Glatirameracetate		2 (14.3)	2 (25)	–
Dimethylfumarate		1 (7.1)	–	–
Teriflunomide		1 (7.1)	–	–
GC		–	2 (25)	5 (62.5)
Rituximab		–	1 (12.5)	–
None		2 (14.3)	3 (37.5)	3 (37.5)

*Age, EDSS, and disease duration are presented as mean ± SD. n.a., not applicable*.

### Purification and Short-Term Culture of Human Monocytes

**Figure 1:**
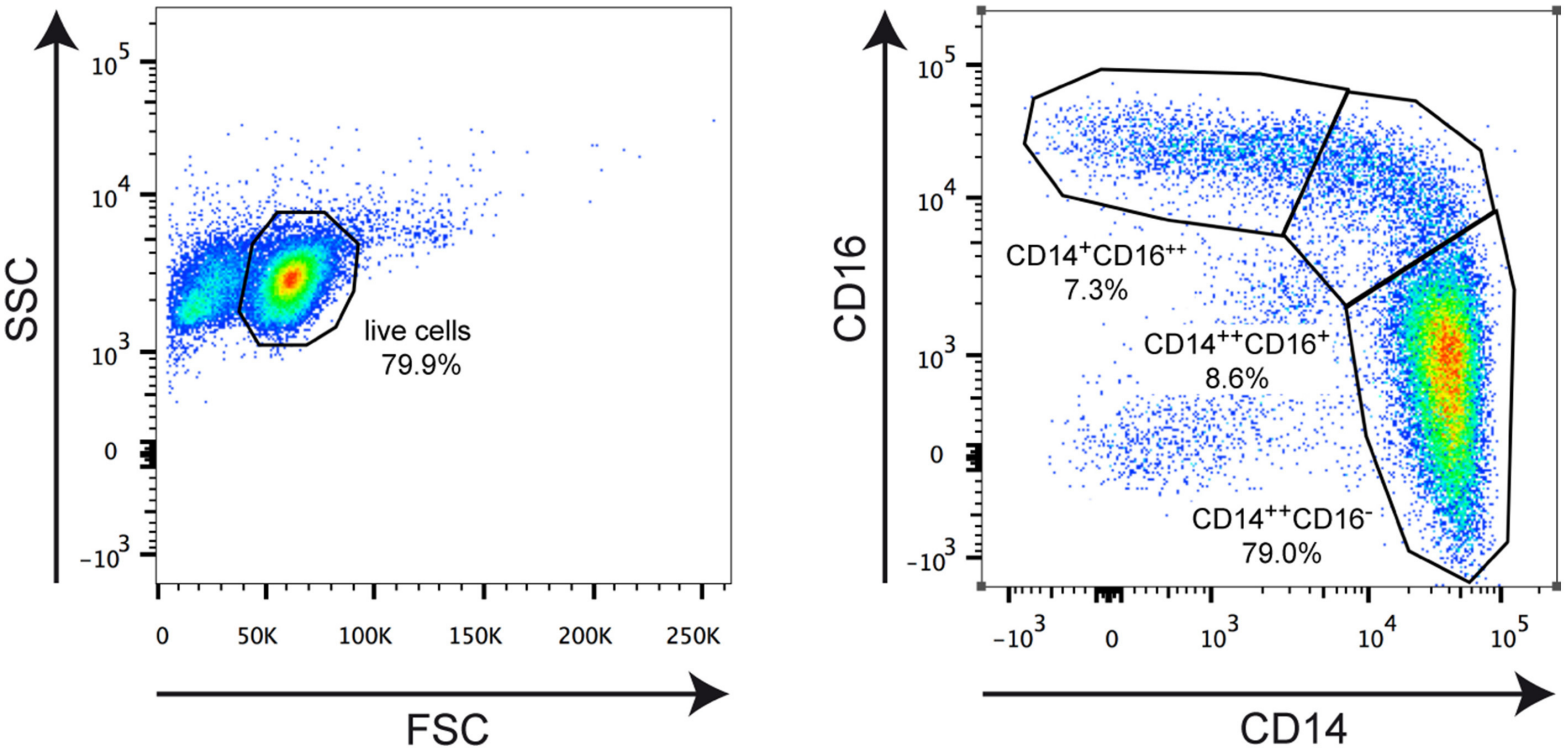
Representative FACS analysis illustrating the applied gating strategy. Monocytes were isolated from an MS patient and stained for CD14 and CD16 surface expression using fluorochrome-conjugated monoclonal antibodies. The left plot depicts the gating for living cells based on forward scatter (FSC) and side scatter (SSC). The right plot shows gating for classical CD14^++^CD16^−^ monocytes, intermediate state CD14^++^CD16^+^ monocytes, and non-classical CD14^+^CD16^++^ monocytes. The borders of the gates and the percentages of cells therein are indicated in each plot.

Peripheral blood lymphocytes were enriched using a lymphoprep gradient (Axis Shield, Oslo, Norway) as described (11), and monocytes were purified with magnetic beads (Stemcell Technologies, Köln, Germany). Purity was assessed on the basis of CD14/CD16 staining by flow cytometry using a FACSCanto II device (BD Biosciences, Heidelberg, Germany), and routinely >95% (Figure 1). Monocytes were analyzed directly or cultured for 3 h in RPMI 1640 medium supplemented with 0.5% fatty acid-free BSA under serum-starved conditions in the presence or absence of 10^−6^ M MP. One portion of the cells was used for RNA isolation and surface marker analyses and the other portion served to assess the migratory capacity.

### Flow Cytometry

Flow cytometric analysis of monocytes was performed as previously described (11). To this end, cells were stained with the following monoclonal antibodies (BioLegend, Uithoorn, The Netherlands) in PBS supplemented with 0.1% BSA and 0.01% NaN_3_: anti-human CD14-PE/Cy7 (clone: HCD14), anti-human CD16-APC/Cy7 (clone: 3G8), anti-human CD163-PE (clone GHI/61), anti-human CD192 (CCR2)-PerCP/Cy5.5 (clone: K036C2), and anti-human CX3CR1-FITC (clone: 2A9-1). Data were acquired on a FACS Canto II device (BD Bioscience) and analyzed using FlowJo® software (Tree Star, Ashland, OR).

### Boyden Camber Assay

After 3 h *in vitro* cultivation with or without MP (see above), 5 × 10^5^ monocytes per well were subjected to a transwell assay using a pore size of 5 μm (Corning Life Sciences, NY, USA) as previously described (11). Cells were allowed to migrate along a gradient of 10 ng/ml CCL2, 10 ng/ml CCL5, or 1 ng/ml CX3CL1 (ImmunoTools, Friesoythe, Germany) for 1 h. The medium in the lower chamber was harvested and the transmigrated monocytes attached to the plate were incubated with 2 mM EDTA in PBS for 20 min at 37°C. Detached cells were scratched off the well bottom and pooled with the harvested medium for analysis. Finally, cells were quantified by flow cytometric analysis using Calibrite Beads (BD Bioscience).

### Quantitative RT-PCR

Quantitative RT-PCR was performed as previously described (11). To this end, total RNA was isolated using the Quick-RNA MiniPrep Kit (Zymo, Irvine, CA) and cDNA was prepared with the iScript Kit (Bio-Rad, Munich, Germany). Quantitative RT-PCR was performed on an ABI 7500 instrument (Applied Biosystems, Darmstadt, Germany) using the SYBR mastermix from the same company. Results were normalized to the mRNA expression of *HPRT* and evaluated using the ΔΔCt method. Primer sequences are depicted in Table 2.

**Table 2:** Primer sequences used for quantitative RT-PCR analysis.

**Gene name**	**Forward primer**	**Reverse primer**
*NR3C1*	AAG AGC AGT GGA AGG ACA GC	CCA GGT TCA TTC CAG CCT GA
*IL1B*	AAC AGG CTG CTC TGG GAT TC	AGT CAT CCT CAT TGC CAC TGT
*CD163*	GGC TTG CAG TTT CCT CAA GA	AGC TGA CTC ATG GGA ATT TTC TG
*CD206*	CGA TCC GAC CCT TCC TTG ACT	AGT ATG TCT CCG CTT CAT GCC
*IL10*	AAG ACC CAG ACA TCA AGG CG	AAT CGA TGA CAG CGC CGT AG
*ARG1*	GGA GTC ATC TGG GTG GAT GC	GGC ACA TCG GGA ATC TTT CCT
*HPRT*	CCT GGC GTC GTG ATT AGT GA	CGA GCA AGA CGT TCA GTC CT

### Statistical Analysis

Data sets were initially subjected to the Shapiro-Wilk normality test to analyze Gaussian distribution. Depending on the results, either a parametric or a non-parametric test was employed, and in the case of matched data, a paired test was used. Accordingly, the experimental groups were compared with a *t*-test, Mann Whitney test, Wilcox matched-pairs signed rank test, or a One-way ANOVA followed by Newman-Keuls Multiple Comparison test as outlined in the figure legends. Analyses were performed with GraphPad Prism software (San Diego, CA). Data are depicted as box-and-whiskers plots showing the minimum, maximum and median, or as the mean ± SEM in all other types of graphs. Levels of significance are as follows: n.s. *p* ≥ 0.05; ^*^p < 0.05; ^**^p < 0.01; ^***^p < 0.001.

## Results

### The Abundance of Classical CD14^++^CD16^−^ Monocytes Is Increased in MS Patients Independently of Disease Activity

**Figure 2:**
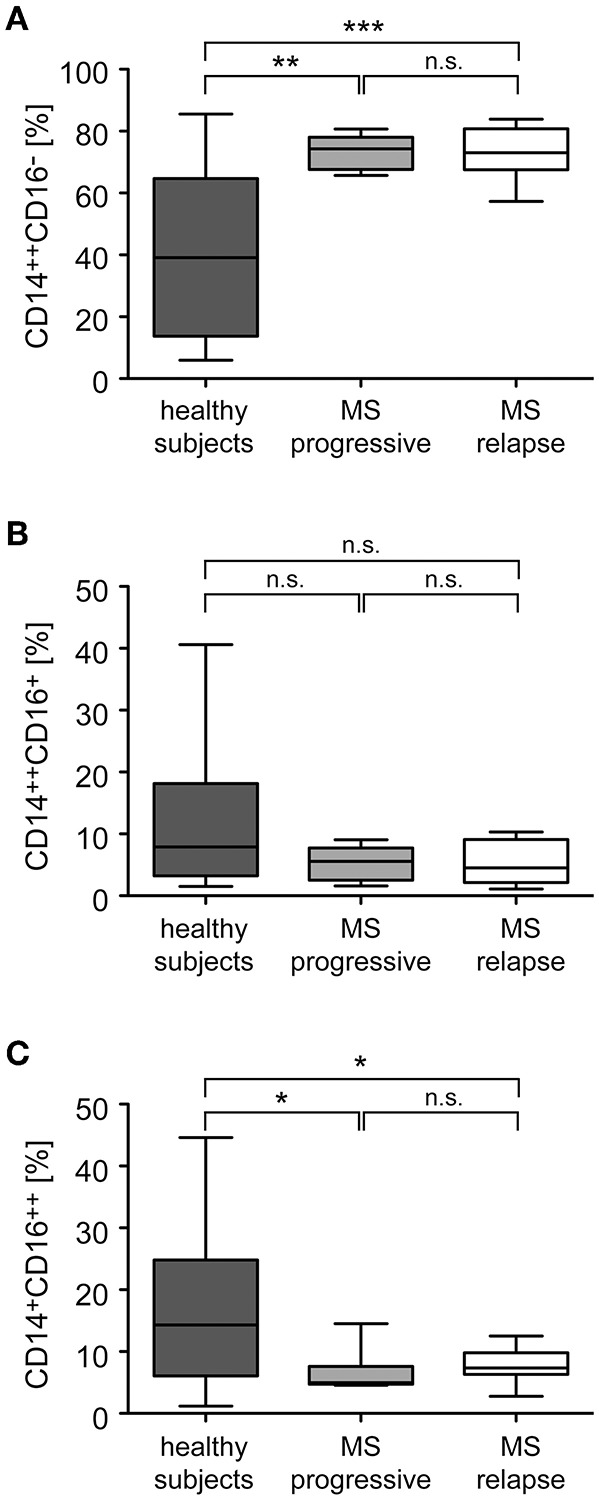
Distribution of monocyte subsets in the peripheral blood of healthy subjects and MS patients with progressive disease or an acute relapse. Monocytes were isolated from the peripheral blood and the percentages of CD14^++^CD16^−^ inflammatory monocytes **(A)**, CD14^++^CD16^+^ intermediate state monocytes **(B)**, and CD14^+^CD16^++^ non-classical monocytes **(C)** were determined by flow cytometry. MS patients were divided into two groups according to their disease activity (progressive, relapse). Data are presented as box-and-whiskers plots showing the minimum, maximum and median; *n* = 20 (healthy subjects), *n* = 8 (MS progressive), *n* = 12 (MS relapse). Statistical analysis was performed using a One-way ANOVA and Newman-Keuls Multiple Comparison test. Levels of significance: n.s. *p* ≥ 0.05; ^*^p < 0.05; ^**^p < 0.01; ^***^p < 0.001.

Monocytes were purified from the peripheral blood of healthy individuals and MS patients and analyzed for the distribution of cellular subsets by flow cytometry (Figure 1). Classical CD14^++^CD16^−^ monocytes were significantly more abundant in MS patients than in healthy control subjects whereas non-classical CD14^+^CD16^++^ monocytes were less frequent in MS patients (Figure 2). In contrast, the percentage of intermediate state CD14^++^CD16^+^ monocytes was unaltered. Noteworthy, these findings are in line with previous reports (19). Furthermore, we did not observe any differences concerning the abundance of monocyte subtypes between MS patients with progressive disease and those undergoing an acute relapse (Figure 2).

### GCs Have Only a Minor Impact on Monocyte Subset Distribution

**Figure 3:**
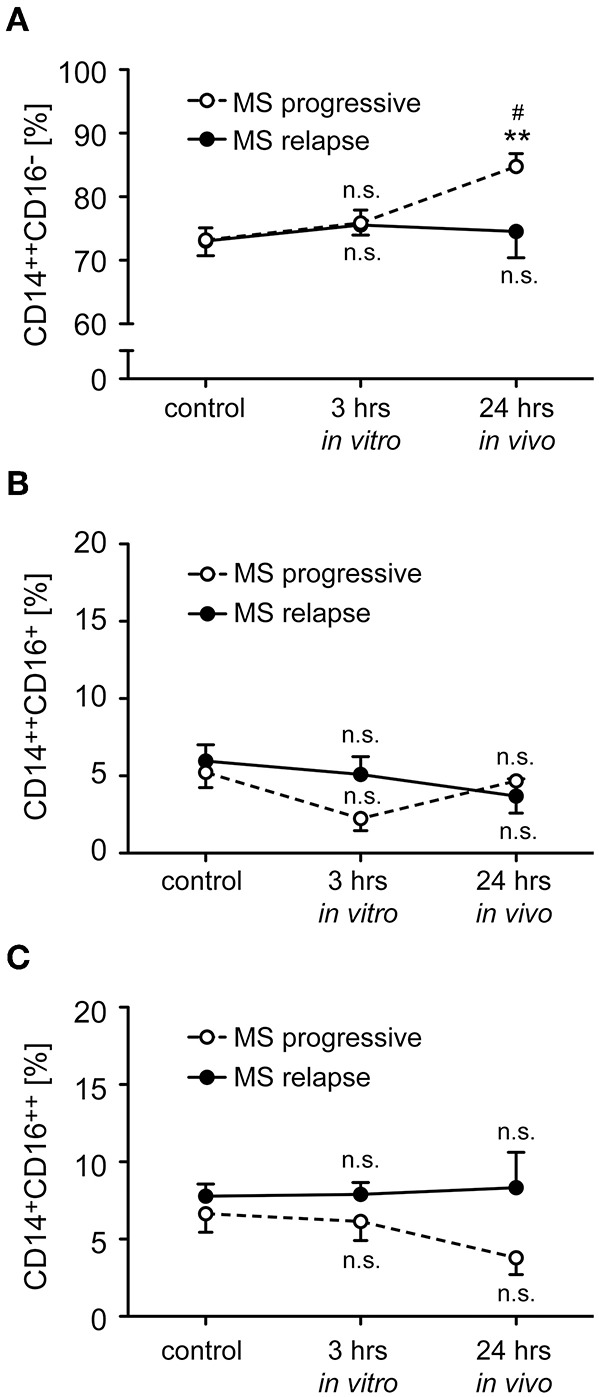
Impact of GC treatment on monocyte subset distribution in MS patients with progressive disease or an acute relapse. Monocytes were isolated from MS patients before MP pulse therapy and incubated for 3 h without (control) or with 10^−6^ M MP *in vitro*. A second blood sample was obtained from the same MS patients 24 h after MP pulse therapy *in vivo*. The percentages of CD14^++^CD16^−^ inflammatory monocytes **(A)**, CD14^++^CD16^+^ intermediate state monocytes **(B)**, and CD14^+^CD16^++^ non-classical monocytes **(C)** were determined by flow cytometry. MS patients were divided into two groups according to their disease activity (progressive, relapse). Data are presented as the mean ± SEM; *n* = 8 (MS progressive), *n* = 12 (MS relapse). Statistical analysis was performed using a One-way ANOVA and Newman-Keuls Multiple Comparison test. Levels of significance: n.s. *p* ≥ 0.05; ^**^p < 0.01 (control vs. 24 h); ^#^*p* < 0.05 (3 vs. 24 h).

Monocytes were isolated from MS patients with progressive disease or an acute relapse before they received a bolus injection of MP. To study short term effects of GCs, the *ex vivo* retrieved cells were incubated for 3 h *in vitro* in the absence (control) or presence of 10^−6^ M MP. In addition, monocytes were isolated from the same MS patients again 24 h after MP pulse therapy to determine long term effects of GC treatment *in vivo*. In the case of patients with an acute relapse, monocyte subset distribution remained unaltered by MP treatment with regard to both short and long term effects (Figure 3). In contrast, we observed an increased frequency of classical CD14^++^CD16^−^ monocytes in patients with progressive disease after long term MP pulse therapy, and a concomitant but non-significant reduction of non-classical CD14^+^CD16^++^ monocytes (Figure 3).

### GCs Induce Monocyte Polarization Toward an Anti-inflammatory M2 Phenotype

**Figure 4:**
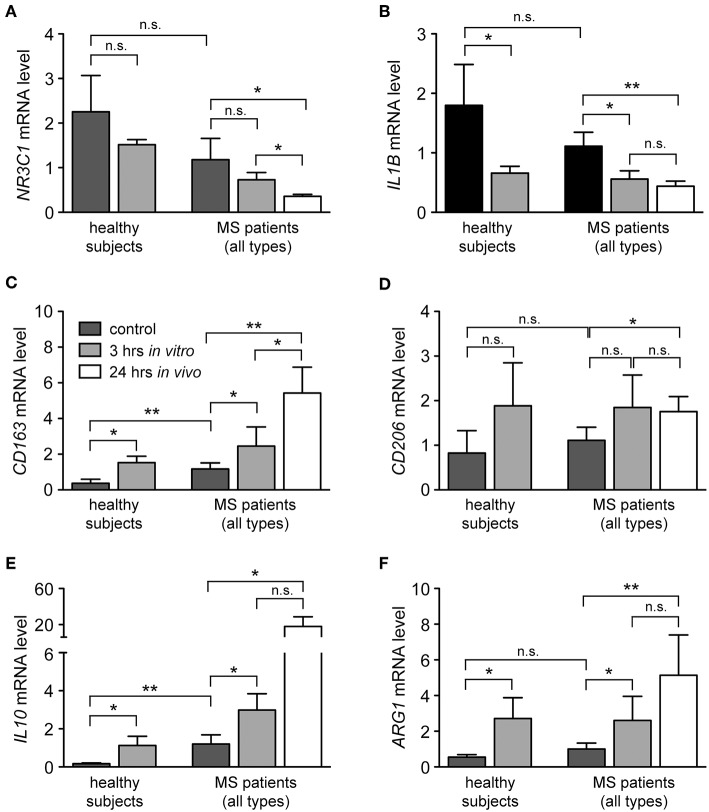
Modulation of the phenotype of monocytes from healthy subjects and MS patients by GCs. Monocytes were isolated from the peripheral blood and cultured without (control) or with 10^−6^ M MP for 3 h *in vitro*. A second blood sample was obtained from the same MS patients 24 h after MP pulse therapy *in vivo*. Thereafter, RNA was prepared and analyzed by quantitative RT-PCR for mRNA levels of *NR3C1* **(A)**, *IL1B* **(B)**, *CD163* **(C)**, *CD206* **(D)**, *IL10* **(E)**, and *ARG1* **(F)**. Gene expression was evaluated using the ΔΔCt method and normalized to *HPRT*. Data are presented as the mean ± SEM; *n* = 6 (healthy individuals), *n* = 9 (MS patients). Statistical analysis was performed using a paired *t*-test (*IL1B*, CD206) or a Wilcox matched-pairs signed rank test (*NR3C1, CD163, IL10, ARG1*). Levels of significance: n.s. *p* ≥ 0.05; ^*^p < 0.05; ^**^p < 0.01.

**Figure 5:**
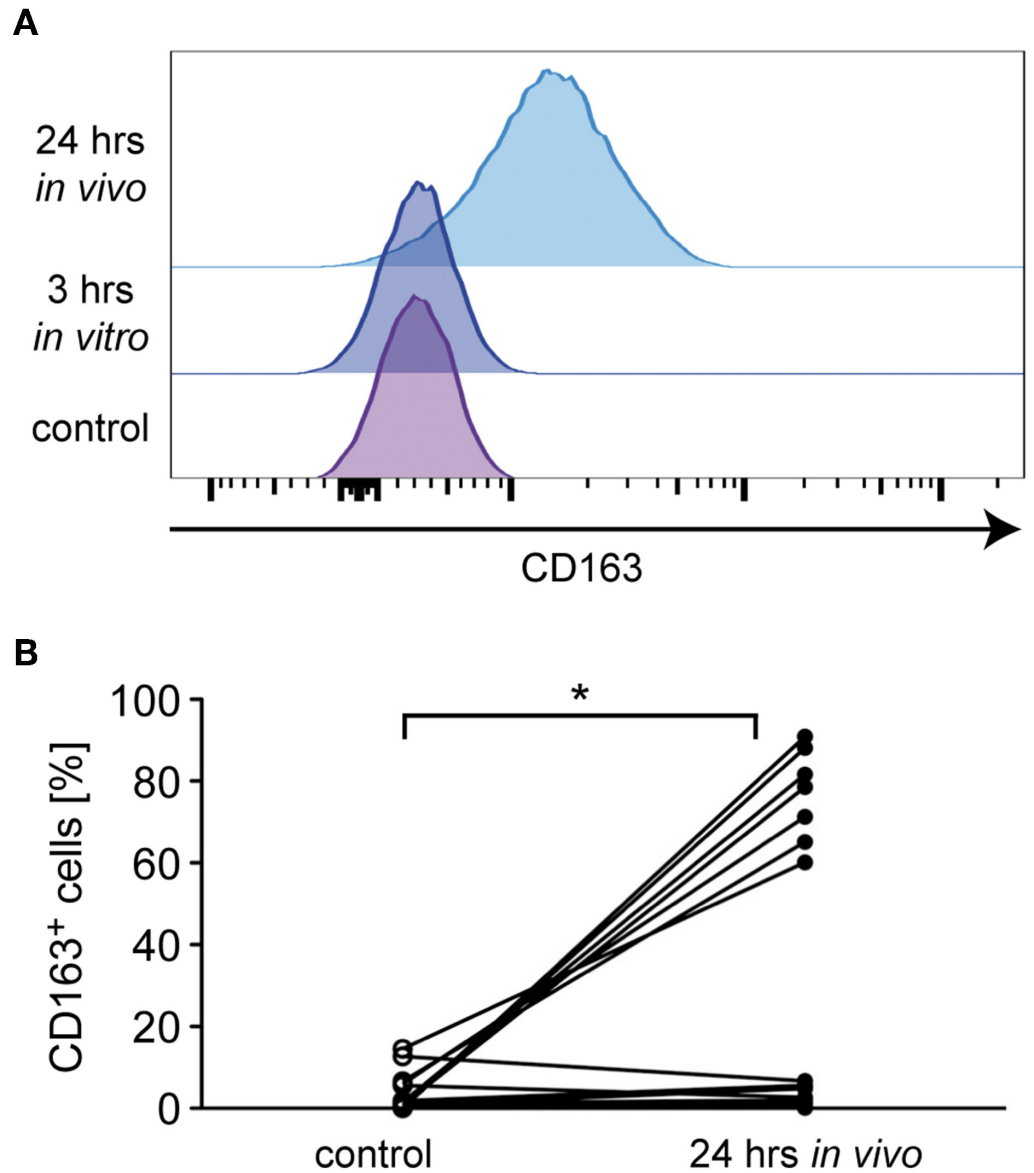
Analysis of monocyte CD163 surface levels in MS patients. Monocytes were isolated from MS patients before MP pulse therapy and cultured without (control) or with 10^−6^ M MP for 3 h *in vitro*. A second blood sample was obtained from the same MS patients 24 h after MP pulse therapy *in vivo*. CD163 surface expression was analyzed by flow cytometry on all cells independently of the CD14/CD16 status. **(A)** Representative stacked histograms are depicted for an MS patient in which CD163 surface levels were upregulated after MP pulse therapy. **(B)** Percentages of CD163^+^ monocytes before (control) and 24 h after MP pulse therapy *in vivo*. The corresponding values for each patient are connected by a line. *n* = 15. Statistical analysis was performed using a Mann Whitney test. Levels of significance: ^*^p < 0.05.

In addition to the classification of monocytes on the basis of cell surface receptors, their phenotype can be characterized by determining their gene expression profile. To this end, we performed an mRNA expression analysis of genes that have been linked to either an M1 or M2 polarization. Monocytes were isolated from healthy subjects and MS patients, incubated with or without 10^−6^ M MP *in vitro* for 3 h and analyzed by quantitative RT-qPCR. In addition, long term GC effects were investigated 24 h after MP pulse therapy *in vivo* (only patients). Initially, we analyzed mRNA levels of *NR3C1*, the gene encoding the GC receptor (GR). *NR3C1* expression did not significantly differ between healthy subjects and MS patients and was reduced by MP treatment as expected (39, 40). However, the latter effect reached statistical significance only in the case of the *in vivo* therapy (Figure 4A). Expression analysis further revealed that mRNA levels of *IL1B*, a pro-inflammatory cytokine that is typical for an M1 polarization of monocytes, were reduced by MP treatment in healthy subjects and MS patients both *in vitro* and *in vivo* (Figure 4B). Concomitantly, the M2 marker genes *ARG1, CD163*, and *CD206* as well as the gene encoding the anti-inflammatory cytokine *IL10* were all increased in monocytes of healthy subjects and MS patients following MP treatment, although the differences were not always statistically significant (Figures 4C–F). It is noteworthy that in general, GC effects were more pronounced after high-dose MP pulse therapy than following *in vitro* culture (Figure 4). Gene expression levels for *CD163* and *IL10* were found to be elevated in MS patients compared to healthy individuals in the steady state (Figure 4). To confirm our results at the protein level, we analyzed surface expression of CD163 as an example by flow cytometry. There were no differences in CD163 levels after short term MP treatment *in vitro*, either for healthy subjects or MS patients (Figure 5A and data not shown). However, 24 h after MP pulse therapy *in vivo*, CD163 surface levels were strongly elevated in a subgroup of MS patients (Figure 5). Interestingly, 6 out of 7 patients in whom CD163 surface expression on monocytes was upregulated were suffering from an acute relapse. Collectively, MP induces a shift toward the anti-inflammatory M2 monocyte phenotype, which is most evident in MS patients receiving high-dose MP pulse therapy.

### GCs Enhance the Migratory Capacity of Monocytes Along Chemokine Gradients

**Figure 6:**
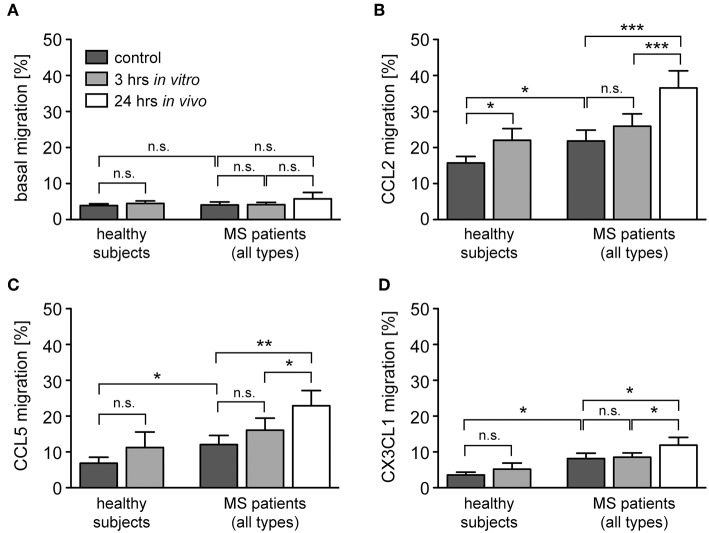
Monocyte migration along chemokine gradients in healthy subjects and MS patients under the influence of GCs. Monocytes were isolated from healthy subjects and MS patients before and after (24 h *in vivo*) MP pulse therapy. Cells were cultured in the absence (control) or presence of 10^−6^ M MP for 3 h *in vitro* and then transferred into the upper part of a Boyden chamber. Basal monocyte migration without a chemokine gradient **(A)** and migration toward a gradient of CCL2 **(B)**, CCL5 **(C)**, or CX3CL1 **(D)** into the lower part of the Boyden chamber were analyzed by flow cytometry and results are depicted as the percentage of transmigrated cells (mean ± SEM). *n* = 19/19/11/9 (healthy subjects), *n* = 13/15/12/17 (MS patients). For statistical analysis, untreated samples were compared to each other using a *t*-test, comparison of untreated vs. MP-treated samples from healthy subjects was performed using a paired *t*-test, and comparison of samples from MS patients to each other was performed using a One-way ANOVA and Newman-Keuls Multiple Comparison test. Levels of significance: n.s. *p* ≥ 0.05; ^*^p < 0.05; ^**^p < 0.01; ^***^p < 0.001.

**Figure 7:**
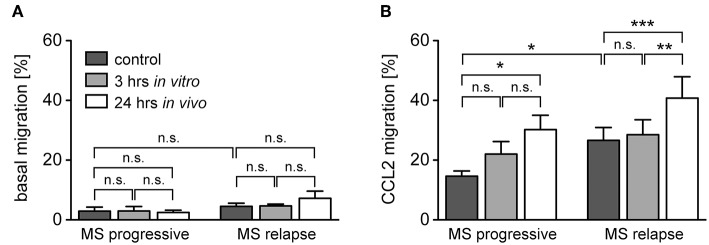
Monocyte migration along a CCL2-gradient in MS patients with progressive disease or an acute relapse under the influence of GCs. The data are the same as in the experiment presented in Figure 4, but the MS patients are now divided into two groups according to their disease activity. Basal monocyte migration without a chemokine gradient **(A)** and migration toward a gradient of CCL2 **(B)** into the lower part of the Boyden chamber were analyzed by flow cytometry and are depicted as the percentage of transmigrated cells (mean ± SEM); *n* = 4/6 (progressive), *n* = 9/9 (relapse). For statistical analysis, untreated samples were compared using a *t*-test and comparison of samples from MS patients to each other was performed using a One-way ANOVA and Newman-Keuls Multiple Comparison test. Levels of significance: n.s. *p* ≥ 0.05; ^*^p < 0.05; ^**^p < 0.01; ^***^p < 0.001.

Transmigration of monocytes across the BBB and infiltration into the meninges and parenchyma is a hallmark of MS and guided by a set of pro-inflammatory chemokines (41). It is against this background that we determined the migratory capacity of monocytes from healthy subjects and MS patients after GC treatment *in vitro* and *in vivo*. The spontaneous basal migration rate of monocytes in the absence of a chemokine gradient was low and independent of disease status and MP treatment (Figure 6A). Expectedly, monocytes migrated toward the chemokines CCL2, CCL5, and CX3CL1, with a higher migratory activity observed for monocytes from MS patients compared to healthy individuals (Figures 6B–D). Short term *in vitro* culture slightly increased the migratory capacity of monocytes retrieved from healthy subjects and MS patients, although significance was missed in most cases (Figures 6B–D). In contrast, *in vivo* MP pulse therapy of MS patients strongly and significantly enhanced the migratory capacity of monocytes in response to all three chemokines (Figures 6B–D). In addition, we further dissected the migratory capacity of monocytes toward CCL2, the chemokine that caused the largest effects, for MS patients according to their individual disease activity. It turned out that the basal migration was the same in both groups, whereas MS patients with progressive disease had a lower CCL2-directed migration than MS patients with an acute relapse (Figure 7). Importantly, the results for monocyte migration toward CCL2 were comparable for both groups with regard to short and long term MP effects (Figure 7B). Furthermore, the same tendency was observed for patients from different MS subtypes (RRMS, SPMS, PPMS), although statistical significance was not reached here due to limited numbers of patients (data not shown). In summary, our data indicate that high-dose MP pulse therapy of MS patients enhances monocyte chemotaxis.

### The Frequency of CCR2^+^ Monocytes and Their CCR2 Surface Expression Levels Are Unaffected by GC Treatment

**Figure 8:**
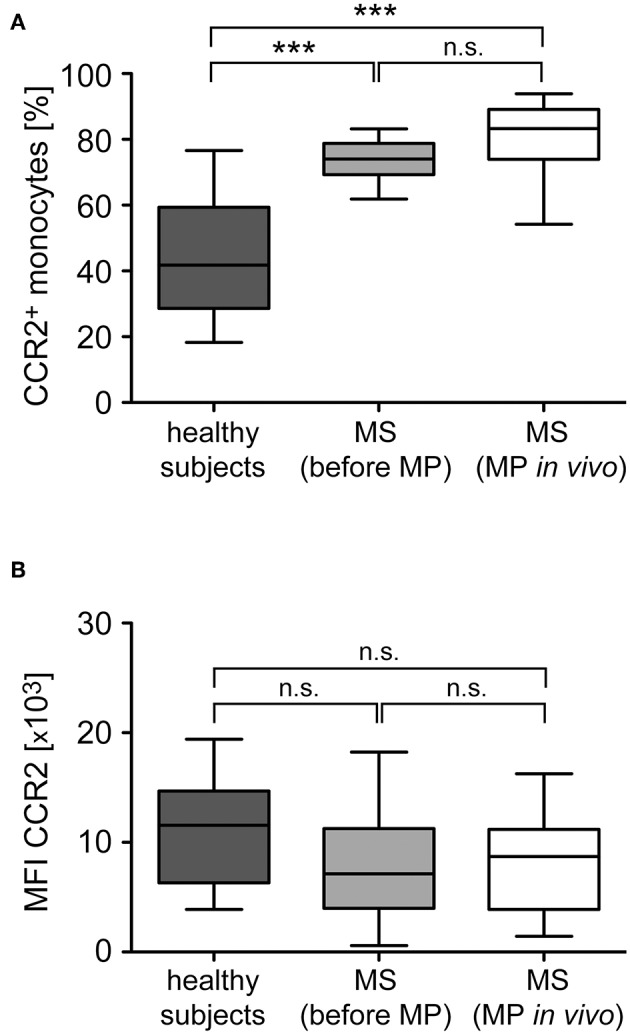
Analysis of CCR2 surface expression levels in monocytes from healthy subjects and MS patients before and after GC treatment *in vivo*. Monocytes were isolated from healthy subjects as well as MS patients before and 24 h after MP pulse therapy *in vivo*. CCR2 surface expression was analyzed by flow cytometry and subsequently the percentage of CCR2^+^ monocytes **(A)** and the surface level of CCR2 based on the mean fluorescence intensity (MFI) were determined **(B)**. Data are presented as box-and-whiskers plots showing the minimum, maximum and median; *n* = 13/22/17. Statistical analysis was performed using a One-way ANOVA and Newman-Keuls Multiple Comparison test. Levels of significance: n.s. *p* ≥ 0.05; ^***^p < 0.001.

Monocyte migration along chemokine gradients depends on the surface expression of the respective receptors as well as intracellular signaling pathways and cytoskeletal rearrangements. To distinguish between these mechanisms, we tested alterations in chemokine receptor expression levels exemplified for CCR2, the receptor of CCL2 which is the chemokine that induced the most robust migration and alteration by GC treatment (Figures 6, 7). The percentage of CCR2^+^ monocytes in MS patients was significantly higher than in healthy subjects (Figure 8A), which is in agreement with their higher percentage of classical inflammatory CD14^++^CD16^−^ monocytes (Figure 2). In contrast, the surface density of this receptor was not significantly changed (Figure 8B). Importantly, MP pulse therapy of MS patients neither altered the abundance of CCR2^+^ monocytes nor the surface expression levels of the receptor (Figures 8A,B), indicating that the increased migration of monocytes toward CCL2 after MP treatment was unrelated to GC effects on the chemokine receptor itself. Notably, CX3CR1^+^ monocytes in MS patients were less abundant than in healthy subjects and unaffected by MP pulse therapy (data not shown), which is also in line with the lower abundance of non-classical CD14^+^CD16^++^ monocytes in MS patients regardless of their treatment (Figure 2).

## Discussion

MS is a complex disease involving multiple interactions between different immune cell populations. Although T cells undoubtedly play a very important role in the pathogenesis of MS, monocytes are implicated in disease pathogenesis too and therefore represent potential therapeutic targets. They can contribute to inflammatory processes by influencing Th17 cell differentiation (30) and impact T-cell activation and differentiation, e.g., by down-regulation of cytokine production or induction of Treg cells at the site of inflammation (37). Although progress has been made in the understanding of these processes (42, 43), the role of different monocyte subsets in autoimmune diseases such as MS remains incompletely understood. Monocytes are rapidly mobilized in large numbers to inflamed sites and also possess T cell-independent effector functions such as phagocytic activity and the secretion of pro-inflammatory cytokines and chemokines. Interestingly, these cells can even be found in the healthy human brain. Especially intermediate state CD14^++^CD16^+^ monocytes are present in the CSF of healthy subjects, where they account for >50% of all monocytes (19), which highlights their importance for the immune surveillance of the CNS. In pathological conditions such as MS, monocytes are found in active and early demyelinating lesions and thus may contribute to the breakdown of the BBB. Nevertheless, depletion of this cell type is not advisable as they can also have beneficial effects in the resolution phase of inflammation and repair processes. For instance, when myeloid cells transduced with the innate immune receptor TREM2 were applied in EAE mice, they created an anti-inflammatory milieu in the CNS resulting in the amelioration of clinical symptoms and reduced structural damage (44). It is further noteworthy that a removal of monocytes would be difficult to achieve because they only have a short half-life of a few days. Selectively employing the anti-inflammatory capacity of monocytes while avoiding a general immune suppression might, however, be favorable for the treatment of autoimmune diseases like MS. In this respect it is relevant that GCs induce a stable anti-inflammatory phenotype in mouse monocytes following their treatment *in vitro* (36). After transfer into recipient mice, such monocytes were found to maintain their polarization and were able to repress T-cell-mediated inflammation even when it was already established (37). Astonishingly, relatively little is known about the mechanisms by which GCs impact this immune cell population during MS pulse therapy, albeit myeloid cells are known to contribute to the pathogenesis of MS and EAE. Expression of the GC receptor in myeloid cells was found to be dispensable for the treatment of EAE with free dexamethasone (7). However, when the GCs were targeted to myeloid cells by encapsulation into liposomes (9) or nanoparticles (10), their effects on monocytes/macrophages turned out to be crucial for their therapeutic efficacy in the same EAE model. In this case, GC treatment resulted in a strong M2 polarization of myeloid cells, which was essential for an amelioration of the disease symptoms (9, 10).

In this study, we report that GCs also have pronounced effects on human monocytes, especially those from MS patients. *In vitro* culture of monocytes from MS patients with MP resulted in an increased gene expression of the M2 markers *ARG1, CD163*, and *CD20*6 and the anti-inflammatory cytokine *IL10*, and the concomitant down-regulation of the mRNA level of the pro-inflammatory cytokine *IL1B*. This effect was even more pronounced after MS patients were subjected to 24 h of MP pulse therapy. Hence, GCs induce an anti-inflammatory M2 monocyte phenotype in MS patients. Surprisingly, we found that the migration of monocytes toward several pro-inflammatory chemokines was enhanced after the exposure to MP *in vitro* and *in vivo*. Presumably, this effect is mostly independent of a modulation of the expression levels of the respective chemokine receptors. Neither the frequency of CCR2^+^ monocytes nor the surface level of this receptor were significantly increased after MS patients underwent MP pulse therapy. Hence it is likely that GCs influence processes other than chemokine receptor levels leading to an enhanced chemotaxis of monocytes. It is noteworthy that we previously described a similar phenomenon for the impact of GCs on the migratory behavior of T cells toward CCL19 and CXCL12, which turned out to be independent of the levels of the respective chemokine receptor as well (11). Therefore, it appears likely that downstream signaling pathways are responsible for the altered migration of monocytes after GC treatment. It has been reported that phospholipase C and phosphokinase C are involved in CCR2 signaling (45), leading to an activation of focal adhesion kinase (FAK) (46). The observation that FAK is phosphorylated in response to GCs in T cells (11) provides a possible explanation for synergistic effects of GCs and CCL2 on monocyte migration. Further support for this notion comes from a report that paxillin, a downstream signaling molecule of FAK, is induced by GCs in human mesenchymal stem cells and thereby enhances their migration (47), and that GCs influence the cytoskeleton of T cells via phospholipase C (48), an effect which could also impact the migratory behavior of monocytes.

Interestingly, frequencies of inflammatory and non-classical monocytes were not substantially influenced by MP treatment although our gene expression analysis revealed a shift toward an anti-inflammatory phenotype under these conditions. Apparently, the cell populations defined by either surface expression of CD14 and CD16 or gene expression of anti-inflammatory molecules are different. It has been shown that human M2 polarized macrophages display a more motile phenotype and migrated more directed and over longer distances toward CCL2 as compared to M1 or M0 macrophages (49). Additionally, it has been hypothesized that the inflammatory chemokine CCL2 might have a beneficial role in MS because its levels are higher in the remission phase than during relapses, although no clear explanation for this phenomenon could be provided (50). This suggests that in phases of disease remission M2 polarized macrophages and monocytes are recruited to the site of inflammation by CCL2, where they promote repair and remyelination. We postulate that the application of GCs enhances this natural repair mechanism by affecting two different aspects of this process. First, GC application promotes M2 polarization of monocytes and second, it enhances the migration of these M2 polarized monocytes toward different chemokines. It is tempting to speculate that under these circumstances anti-inflammatory monocytes already reach the CNS at time points when natural repair mechanisms have not yet been initiated, thereby accelerating and optimizing the repair process and facilitating the remission of the disease.

Although T cells are still widely considered to be the major target cells of MP pulse therapy, resulting in changes in cytokine expression, adhesion molecule expression, migration and apoptosis (51), some effects of GCs on myeloid cells have already been described in the past. For instance, the phagocytic potential of human monocytes was enhanced by the incubation with dexamethasone *in vitro* (52). Interestingly, enhanced phagocytosis of macrophages *in vitro* is associated with an M2 polarization (53, 54). Furthermore, high-dose MP pulse therapy resulted in a decreased frequency of monocytes producing IL-8, which is typical for the inflammatory CD14^++^CD16^−^ subset (55). While we did not observe a change in the frequency of inflammatory or non-classical monocytes 24 h after MP pulse therapy, it is noteworthy that the aforementioned decrease of IL-8-producing monocytes was described 5 days after treatment, suggesting that such a change might be evident only at a later time point. In addition, GC treatment of EAE in mice resulted in a reduced expression of beta-arrestin-1 and enhanced mRNA levels of *A1AR* (56), which is thought to regulate cytokine expression and release and NO production in myeloid cells (57). In fact, mRNA levels of cytokine genes were at least partially affected by GCs in our study: we observed a reduced expression of the pro-inflammatory cytokine *IL1B* and an increased expression of the anti-inflammatory cytokine *IL10*. Interestingly, previous reports indicated that IL-6 levels were not changed by GCs in human monocytes (36), which is in contrast to mouse monocytes (58). The reason for this species difference, however, is unclear. Furthermore, analysis of human monocyte-derived dendritic cells showed that GCs induced IL-10 secretion *in vitro* (59), and analysis of human monocyte-derived macrophages revealed that GCs repressed IL-6 and TNFα responses induced by LPS stimulation *in vitro* (60). Collectively, these findings are in line with our finding that GC treatment modulates cytokine expression by human monocytes.

Our data suggest that CCL2 is the chemokine that controls monocyte migration into the CNS to a higher degree compared to the other chemokines tested in this study. The migration rate of untreated monocytes from healthy subjects and MS patients toward CCL2 was higher compared to CCL5 and CX3CL1, which confirms previous data also showing higher migration rates of human monocytes toward CCL2 in comparison to CX3CL1 (19). Hence, it does not come as a surprise that CCL2-directed migration is also the predominant target of GCs in the context of chemotaxis. Still, it is somewhat contradictory at first sight that chemotaxis toward an inflammatory chemokine is increased by GCs rather than decreased. We believe that this observation needs to be interpreted in the light of the concurrent phenotypic changes that lead to an anti-inflammatory polarization of monocytes. It appears that GCs promote the infiltration of those monocytes into the CNS that are able to terminate inflammation and initiate repair processes, thus contributing to an amelioration of disease symptoms after MP pulse therapy of MS patients. Of note, the occurrence of anti-inflammatory activity of myeloid cells in inflammatory CNS diseases has been reported previously (61, 62) but the exact mechanisms remained incompletely understood.

In summary, GCs exert marked effects on monocytes from MS patients, which could in part explain the therapeutic efficacy of MP pulse therapy. Apparently, these effects are achieved by a combination of M2 polarization and enhanced chemotaxis and certainly play an important role in addition to the well-described impact of GCs on T-cell function. Therefore, GCs should not only be considered as T-cell suppressors but also as modulators of myeloid cells in MS therapy.

## Data Availability

The raw data supporting the conclusions of this manuscript will be made available by the authors, without undue reservation, to any qualified researcher.

## Ethics Statement

This study was carried out in accordance with the recommendations of the local ethics committee of the University Medical Center Göttingen with written informed consent from all subjects, in accordance with the *Declaration of Helsinki*.

## Author Contributions

HF performed and analyzed most of the experiments, and wrote the manuscript. TF performed and analyzed experiments. HP provided blood samples of MS patients. HR designed the project, analyzed experiments, and wrote the manuscript. FL designed the project, analyzed experiments, and wrote the manuscript.

### Conflict of Interest Statement

The authors declare that the research was conducted in the absence of any commercial or financial relationships that could be construed as a potential conflict of interest.
